# Healing of a Large Periapical Lesion Using Non-Surgical Root Canal Retreatment: A Case Report

**DOI:** 10.7759/cureus.69586

**Published:** 2024-09-17

**Authors:** Haya Alyousef, Amani A Almohaimeed

**Affiliations:** 1 Endodontics, Prince Sultan Military Medical City, Riyadh, SAU

**Keywords:** cone-beam computed tomography (cbct), effective disinfectant protocol, large periapical lesion, non-surgical endodontic treatment, single-visit endodontic treatment, teeth requiring non-surgical endodontic treatment

## Abstract

This case report highlights the successful healing of a large periapical lesion through non-surgical root canal retreatment. A 29-year-old male patient presented with a significant radiolucency associated with teeth #21 and #22, initially treated non-surgically. Despite the lesion's size, the treatment, which included thorough canal disinfection and obturation, led to substantial healing. A follow-up cone-beam computed tomography (CBCT) scan after one year confirmed the buccal cortical bone reformation and improvement in the incisive canal area except for the apical region of #21. Subsequently, root canal retreatment was performed for #21. Complete healing was achieved after two years, demonstrating that even extensive periapical lesions can be effectively treated with non-surgical endodontic retreatment, avoiding invasive surgical intervention.

## Introduction

Multiple factors can contribute to pulpal tissue infection, including caries and trauma resulting in necrosis. Most bacteria in the oral microbial flora possess the potential to infiltrate the root canal system both during and after pulp necrosis, thereby contributing to the initiation and progression of root canal infection that results in periapical inflammation. However, the bacterial composition found within infected root canals is more restricted than the members of the oral microbiota [1]. Microbial synergy of different species is a significant virulence factor contributing to a larger periapical lesion size [2]. Once bacteria and their by-products infiltrate the periapical tissue, they activate the host's immune reaction and acquire sophisticated mechanisms to survive the host's defense, thereby enhancing their pathogenicity [1]. The majority of periapical lesions can be classified as either dental granulomas (48.0%), radicular cysts (42.0%), or abscesses (1.2%) [3]. It is well known that despite the advances in imaging technologies, such as cone-beam computed tomography (CBCT), radiography has limitations in determining the nature and the definitive diagnosis of the periapical lesion and its inability to differentiate between granulomas and cystic lesions [4]. Surgical biopsy and histopathological examination remain the established methods for distinguishing between radicular cysts and granulomas.

The rationale of endodontic treatment is to eliminate infection, remove bacteria, debris, and pulp tissue remnants, prevent microorganisms from contaminating or re-contaminating the root and/or periradicular tissues by shaping the root canal to ensure adequate disinfection and facilitate proper obturation, ensure complete sealing of the canal to prevent contamination and ultimately prevent subsequent recontamination of the obturated root canals [5]. Therefore, the inability to meet these standards results in failure of root canal therapy or persistent inflammation and infection. However, even with adherence to strict protocols, apical periodontitis may persist radiographically as asymptomatic apical radiolucencies due to the complexity of the root canal system or extraradicular factors present within inflamed periapical tissues, which can interfere with the healing process [6].

Additionally, the possibility of cystic lesions increases by up to 100% when the radiographic lesion size is 200 mm^2^ or greater [7]. In this occasion, the lesion can be treated non-surgically if it is in direct communication with the root canal system (apical pocket cyst or bay cyst) and responds favorably to treatment. On the contrary, surgical intervention can be the treatment of choice if the lesion is separate from the apex and has an intact epithelial lining (apical true cyst) [8].

With recent advancements in treatment techniques, along with the development of new instruments and biocompatible materials, the healing of periradicular lesions has greatly improved, contributing to higher success rates [9,10]. Therefore, non-surgical root canal treatment has been suggested as the initial approach to treating large periapical lesions without surgical intervention.

The aim of this case report is to recommend non-surgical endodontic retreatment to treat lesions of endodontic inflammatory origin, irrespective of the size of the lesion.

## Case presentation

A 29-year-old man with a noncontributory medical history was classified as American Society of Anesthesiologists (ASA) I according to the ASA physical status classification. The patient was referred to the endodontic clinic to treat a large radiographic radiolucency found apically to #21 and #22. The patient reported no pain or any previous dental trauma. He had a history of nonsurgical root canal treatment five years earlier for tooth #21, and no previous dental interventions were done for tooth #22. Extra-oral examination showed no facial swelling. An intra-oral examination revealed no signs of periodontal involvement, which was confirmed by mobility tests and periodontal probing. Thermal and electric pulp testing was performed for all upper anterior teeth, and all responded normally except for teeth #21 and #22. No pain was reported on percussion and palpation of all examined teeth. The clinical test revealed that tooth #22 is necrotic with asymptomatic apical periodontitis, and tooth #21 was previously treated with asymptomatic apical periodontitis.

**Figure 1:**
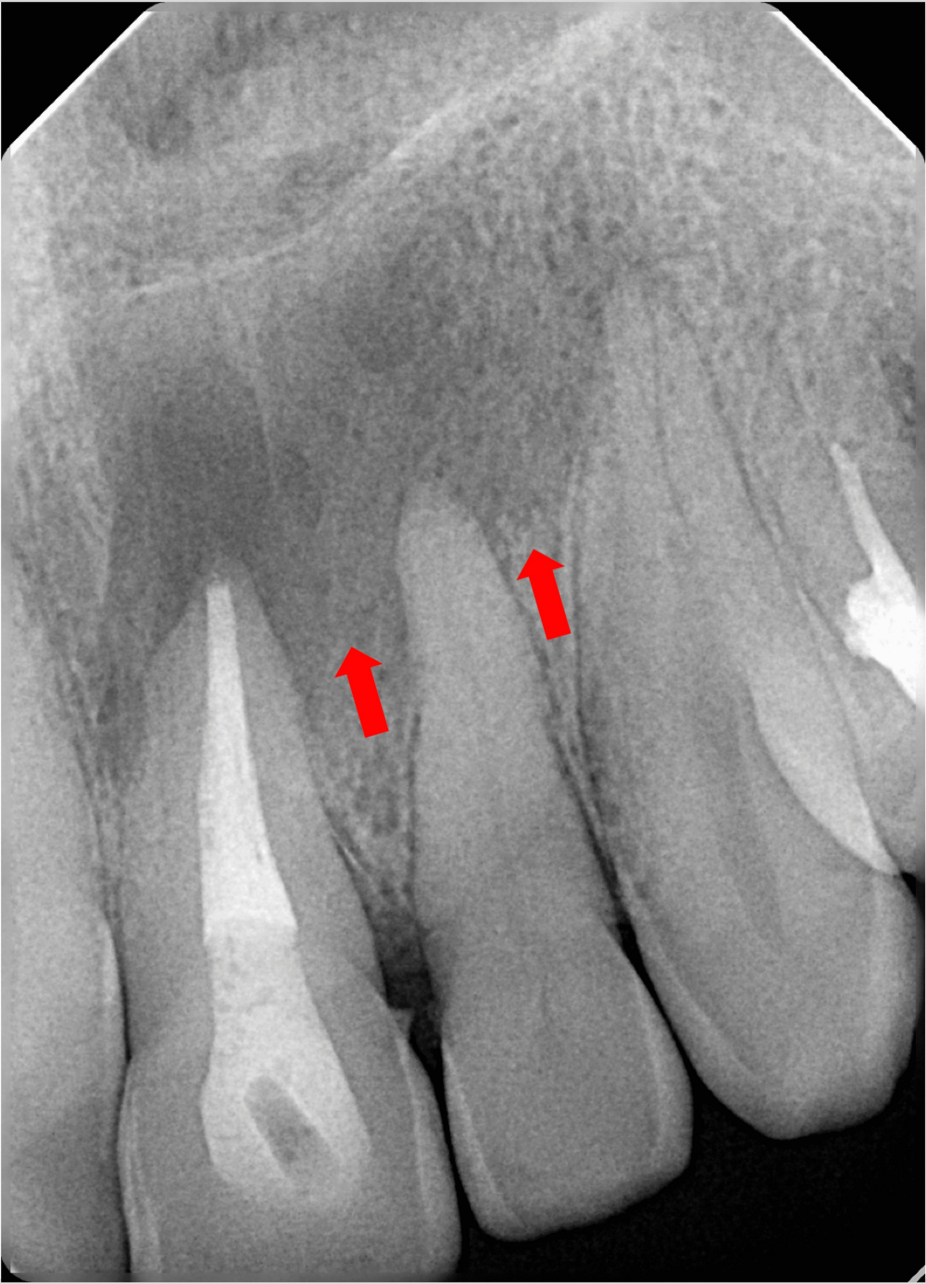
Periapical radiograph demonstrating large peri-apical lesion in the upper anterior region related to teeth #21 and #22.

The periapical radiograph (Figure 1) demonstrated a large non-corticated radiolucent lesion with ill-defined margins around the apex of #21 and #22. Tooth #21 had a radiopaque filling material extending in the root canal space that was within 1-2 mm of the radiographic apex.

**Figure 2:**
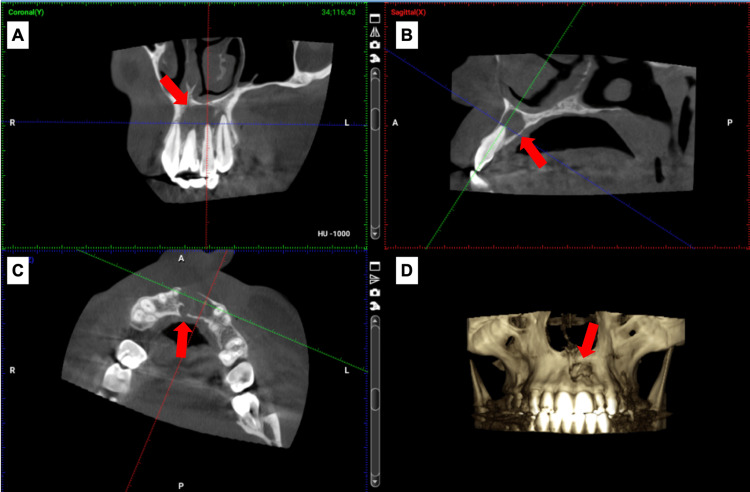
(A) Coronal view; mesiodistal extent of the lesion. (B) Sagittal view; buccolingual extent. (C) Axial view shows resorption of buccal cortical bone and the incisive canal (D) 3D reconstruction. (Red arrows) The lesion location.

CBCT with a limited field of view was used to assess the extent of periradicular radiolucency (Figure 2). CBCT revealed a low-density area (mesiodistal dimension 15 mm) extending from the mesial side of #21, reaching the mesial side of #23 in the largest diameter and (buccolingual dimension 10 mm) shows resorption of buccal cortical bone and also part of incisive canal.

Treatment options were discussed with the patient, starting from the most conservative approach, which is non-surgical root canal treatment of tooth #22 and follow-up to observe the healing for both teeth ( #22 and #21) and consider non-surgical root canal retreatment of tooth #21 if there was no sign of healing. The second option is endodontic microsurgery after root canal treatment of tooth #22. The last option is extracting teeth involved in the lesion and planning for an implant-supported prosthesis. 

Afterward, the decision was made by the patient to non-surgically treat tooth #22 and follow up to assess the healing of the periapical lesion; if the lesion does not subside or reduces in size in a follow-up visit, a nonsurgical root canal retreatment will be performed on tooth #21.

**Figure 3:**
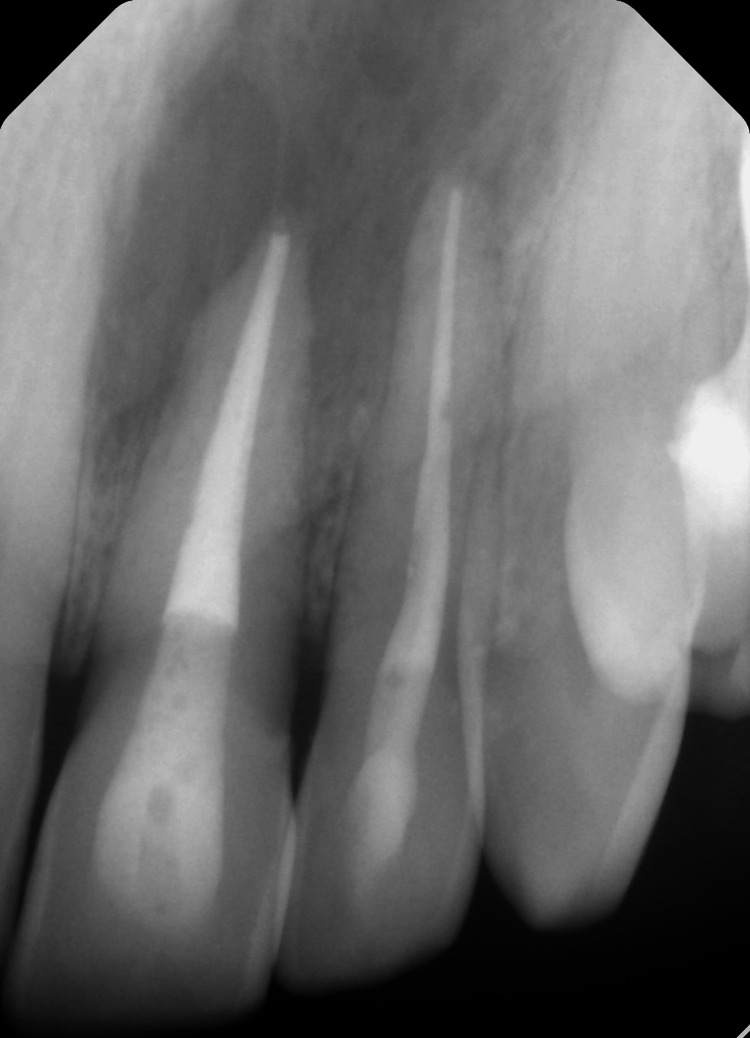
Immediate post-operative radiograph.

The patient provided written informed consent for treatment. Tooth #22 was anesthetized using 2% lidocaine with epinephrine (1:100,000), and a rubber dam was applied. Following access cavity preparation with a diamond bur TF-13C and utilizing a dental operating microscope (DOM), no bleeding was inspected, which confirmed the initial diagnosis. Instrumentation was performed using ProTaper Gold (Dentsply Sirona, Charlotte, NC), reaching F2 and enlarging of apical preparation with profile #35/0.04. Copious irrigation with 5.25% NaOCl and final irrigation with 17% EDTA, NaOCl, and EDTA irrigants were energized with a size 25 EndoActivator (Dentsply Sirona, Charlotte, NC) for one minute. The canal was dried with paper points and obturated using a warm vertical compaction technique with AH Plus (Dentsply Sirona, Charlotte, NC) sealer. The canal was sealed with a double seal using Cavit (3M, St. Paul, MN) and glass ionomer cement (GIC) (Figure 3).

**Figure 4:**
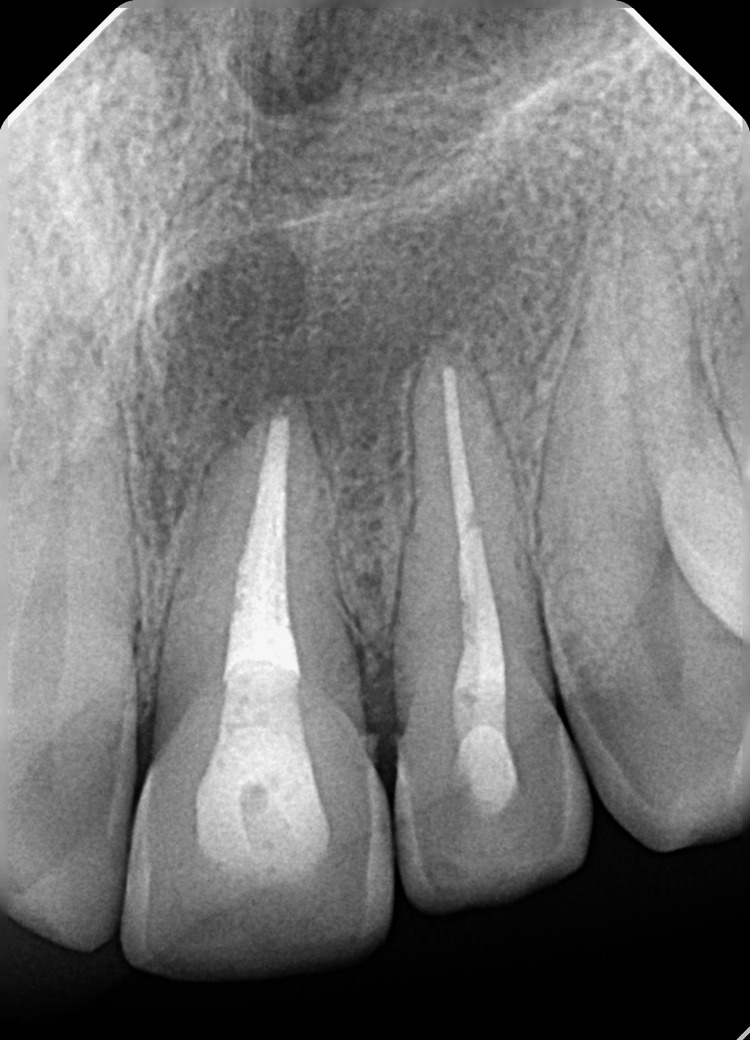
One-year follow-up.

The patient returned for follow-ups after one year (Figure 4). The patient was asymptomatic.

**Figure 5:**
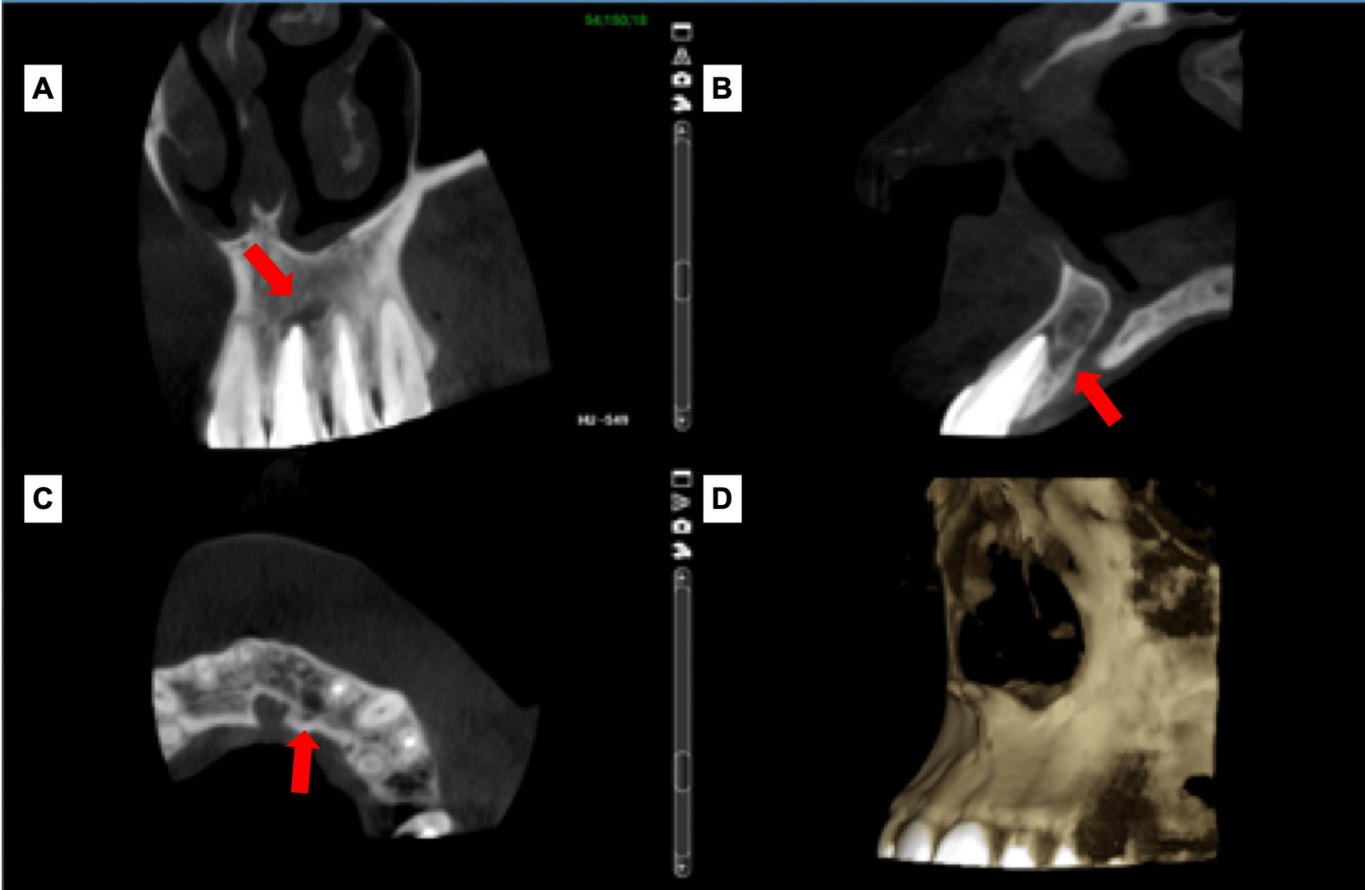
(A) Coronal view, (B) sagittal view, and (C) axial view: healing of the lesion, reformation of buccal cortical bone and incisive canal, except around the apical area of tooth #21. (D) 3D reconstruction.

A new CBCT scan was requested (Figure 5) and revealed healing of the lesion that was presented as reduced radiolucency (reformation of buccal cortical bone and incisive canal) except around the apical area of tooth #21. Non-surgical root canal retreatment was performed for tooth #21 by another dentist following the same protocol.

**Figure 6:**
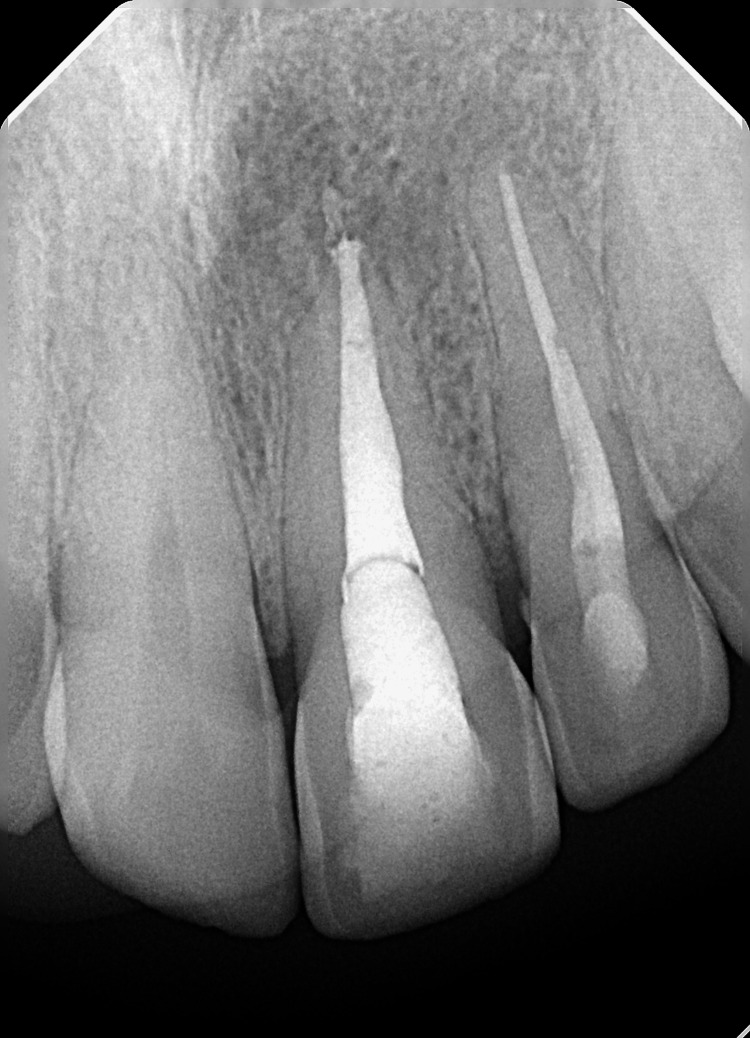
Two-year follow-up periapical radiograph showing complete lesion healing.

**Figure 7:**
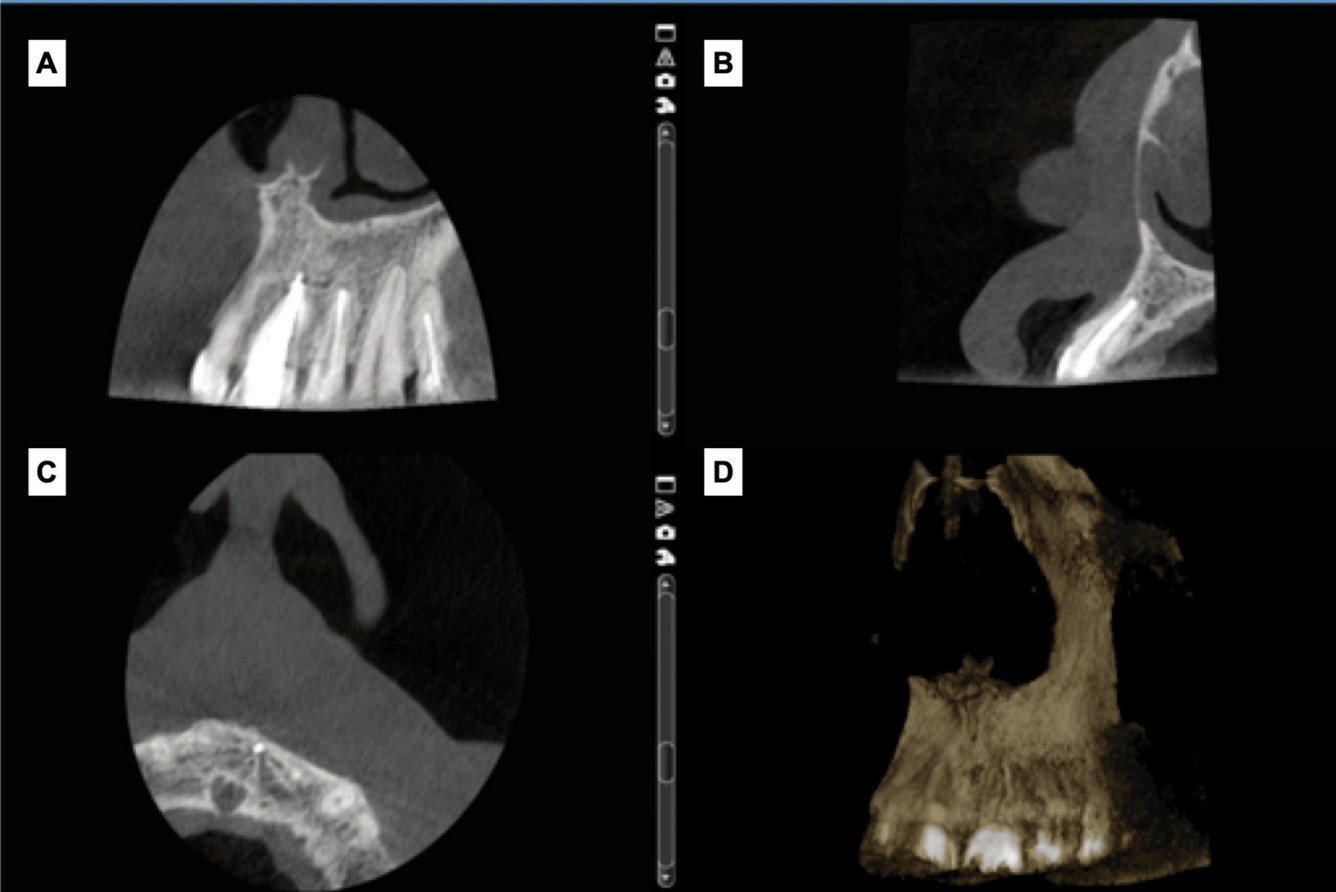
Two-year follow-up using cone-beam computed tomography: healing of the lesion.

A recall after two years using periapical radiographs (Figure 6) and a CBCT scan (Figure 7) showed complete healing.

## Discussion

Root canal treatment is based primarily on the healing of periapical lesion by removal of microbial infection from the complex root canal system. Irrigants aid in reducing the microbial flora of infected canals, and if a tissue-solvent solution is used, it can dissolve necrotic tissues [11].

Several adjunctive methods can be used to facilitate the performance of endodontic treatment, such as magnification tools such as loupes and DOMs, as well as advanced radiographic techniques such as CBCT.

The use of magnification during endodontic procedures, particularly the DOMs, provides enhanced visualization of the operating field, allowing for better discrimination of anatomic details, facilitates better control of instruments and placement of dental materials, and allows for improved detection and management of obstructions, anatomic variations, or fractures [12].

In the present case report, CBCT was utilized as a diagnostic tool initially and also during the subsequent follow-up visit to measure and monitor the healing of periapical lesion and evaluate the success of endodontic treatment.

It has been well-documented in the literature that the pre-operative presence of a lesion adversely affects success [13]. Significant associations between the outcome of initial root canal treatment and the presence of a pre-operative periapical lesion on the healing rate after initial treatment have been observed. With the presence of a periapical lesion, the healing rate is estimated to be 10% lower than in the absence of a periapical lesion [14]. The size of the apical lesion might also affect the outcomes of endodontic surgery, with larger lesions being linked to less favorable healing. A 21% decrease in success was reported for teeth with greater than 5-mm-diameter lesions preoperatively, compared with those with lesions less than 5 mm in diameter [15].

On the other hand, another prospective study reported that the periapical lesion size had no significant effect on the success rate of non-surgical root canal treatment [16]. Single-visit root canal treatment has become a common practice and offers several advantages, such as a reduced flare-up rate [17]. Single-visit root canal treatment was slightly more effective than multiple visits, i.e., 6.3% higher healing rate. However, the difference in healing rate between these two treatment regimens was not statistically significant [18]. The advantages of employing noninvasive nonsurgical treatment as an initial approach, even for large extensive periapical lesions, are of great value, including reduced psychological stress, greater patient acceptance, and predictable excellent outcome, in addition to avoiding the risk of damaging anatomical vital structures associated with surgical intervention and pain and discomfort after the procedure. As in this present case, complete resolution of the periapical lesion was accomplished in a single visit with strict adherence to aseptic procedures under DOM and verified with the use of CBCT.

## Conclusions

This case report demonstrates the efficacy of non-surgical endodontic retreatment in managing large periapical lesions of endodontic origin. Despite the complexity of root canal systems and the challenges posed by extensive lesions, strict adherence to aseptic protocols, advanced imaging techniques like CBCT, and modern endodontic tools, such as the DOM, can lead to successful outcomes without the need for surgical intervention. The complete resolution of the periapical lesion in this case supports the recommendation that non-surgical root canal treatment should be considered as the initial approach, even for extensive lesions, before resorting to surgery. This approach not only reduces patient discomfort and stress but also minimizes the risk of complications associated with surgical procedures, contributing to higher rates of successful healing.
